# Multi-Locus GWAS for Grain Weight-Related Traits Under Rain-Fed Conditions in Common Wheat (*Triticum aestivum* L.)

**DOI:** 10.3389/fpls.2021.758631

**Published:** 2021-10-21

**Authors:** Vijay Gahlaut, Vandana Jaiswal, Harindra S. Balyan, Arun Kumar Joshi, Pushpendra K. Gupta

**Affiliations:** ^1^Department of Genetics and Plant Breeding, Chaudhary Charan Singh University, Meerut, India; ^2^Council of Scientific & Industrial Research-Institute of Himalayan Bioresource Technology, Palampur, India; ^3^International Maize and Wheat Improvement Center (CIMMYT), New Delhi, India; ^4^Borlaug Institute for South Asia (BISA), New Delhi, India

**Keywords:** wheat, drought stress, multi-locus-GWAS, stress tolerance index, SNP joint effect

## Abstract

In wheat, a multi-locus genome-wide association study (ML-GWAS) was conducted for the four grain weight-related traits (days to anthesis, grain filling duration, grain number per ear, and grain weight per ear) using data recorded under irrigated (IR) and rain-fed (RF) conditions. Seven stress-related indices were estimated for these four traits: (i) drought resistance index (DI), (ii) geometric mean productivity (GMP), (iii) mean productivity index (MPI), (iv) relative drought index (RDI), (v) stress tolerance index (STI), (vi) yield index, and (vii) yield stability index (YSI). The association panel consisted of a core collection of 320 spring wheat accessions representing 28 countries. The panel was genotyped using 9,627 single nucleotide polymorphisms (SNPs). The genome-wide association (GWA) analysis provided 30 significant marker-trait associations (MTAs), distributed as follows: (i) IR (15 MTAs), (ii) RF (14 MTAs), and (iii) IR+RF (1 MTA). In addition, 153 MTAs were available for the seven stress-related indices. Five MTAs co-localized with previously reported QTLs/MTAs. Candidate genes (CGs) associated with different MTAs were also worked out. Gene ontology (GO) analysis and expression analysis together allowed the selection of the two CGs, which may be involved in response to drought stress. These two CGs included: TraesCS1A02G331000 encoding RNA helicase and TraesCS4B02G051200 encoding microtubule-associated protein 65. The results supplemented the current knowledge on genetics for drought tolerance in wheat. The results may also be used for future wheat breeding programs to develop drought-tolerant wheat cultivars.

## Introduction

Wheat (*Triticum aestivum* L.) is one of the major cereal crops and contributes about 30% (760 million tons) of world grain production (Food Agriculture Organization of the United Nations, 2021). However, the rate of increase in annual wheat production has recently decreased from 3% during the 1970's and 1980's to 0.9% in recent years causing concern. This rate must increase to ~2% to meet the projected demand of 50–60% additional wheat by 2050 (Ray et al., 2013). This may be challenging owing to a variety of abiotic stresses that impact wheat yield. Among the abiotic stresses, drought is the most important causing yield losses of up to 40% (Zampieri et al., 2017). Recently, an assessment by the UN's Intergovernmental Panel on Climate Change (IPCC) predicted that the global surface temperature would increase by 1.5°C in the next 20 years (by 2040), causing extreme drought in several wheat growing regions including South Asia (IPCC, 2021) which inhabits one-fourth of the global population and where wheat is a lifeline for millions. In most parts of India, wheat is already being grown under restricted (one or two) irrigation (Joshi et al., 2007a). Current trends indicate that such regions may further expand due to climate change (Joshi et al., 2007b; Kumar et al., 2012). Therefore, understanding the genetic systems that provide tolerance to drought stress is a priority for wheat breeding to sustain wheat production and productivity.

Traits associated with tolerance to water stress are complex having polygenic control, low heritability, and large genotype × environment interaction. This situation worsens, when drought is associated with other biotic and abiotic stresses (Fleury et al., 2010), making it difficult to dissect the genetic control of drought tolerance. Multi-locus genome-wide association study (ML-GWAS) is a powerful approach to deal with this problem. The approach has already been successfully utilized to dissect the genetic architecture associated with important agronomic and quality traits in several crops, such as maize (Zhang et al., 2018; Zhu et al., 2018; An et al., 2020), rice (Cui et al., 2018; Liu et al., 2020), barley (Hu et al., 2018), cotton (Li et al., 2018; Su et al., 2018), soybean (Ziegler et al., 2018), and foxtail millet (Jaiswal et al., 2019). In wheat also, ML-GWAS has been used to identify genomic regions associated with different agronomic and yield associated traits (Jaiswal et al., 2016; Ward et al., 2019; Hanif et al., 2021; Malik et al., 2021a; Muhammad et al., 2021), grain architecture-related traits (Schierenbeck et al., 2021), spike-layer uniformity-related traits (Malik et al., 2021b), potassium use efficiency (Safdar et al., 2020), nutrient accumulation (Bhatta et al., 2018; Kumar et al., 2018; Alomari et al., 2021), disease resistance (Cheng et al., 2020; Habib et al., 2020; Tomar et al., 2021), and salinity tolerance (Chaurasia et al., 2020).

Only two ML-GWAS are available for wheat, where MTAs were reported for agronomic traits across drought stress environments (Gahlaut et al., 2019; Li et al., 2019). The present study is a continuation of one of these studies (Gahlaut et al., 2019), where 19 MTAs associated with yield and related traits under drought were reported. The other study involved the use of 277 wheat accessions leading to the identification of an important QTL on chromosome 6A for grain yield under drought (Li et al., 2019). We speculate that these two studies did not exhaust the possibility of identification of all possible MTAs, thus warranting further studies to facilitate the identification of more robust MTAs for marker-assisted selection (MAS). Therefore a ML-GWAS was conducted using an association panel of 320 spring wheat accessions to detect novel MTAs and important CGs for drought stress tolerance.

## Materials and Methods

### Plant Material and Field Experiment

A panel of 320 spring wheat genotypes [hereafter called wheat association mapping (WAM) panel], representing a world collection from 28 different countries, was used. This diverse panel was procured from the International Maize and Wheat Improvement Center (CIMMYT), Mexico [for details, see Gahlaut et al. (2019)]. The WAM panel was raised under irrigated (IR) and rainfed (RF) conditions each at two different locations: (i) Meerut [North India; (28°0.97′N 77°0.74′E)] and (ii) Powarkheda (Central India; 22°0.07′N 73°0.98′E) in crop season 2011–12 and 2012–13, respectively; thus providing following four environments: Meerut IR (E1), Meerut RF (E2), Powarkheda IR (E3), and Powarkheda RF (E4). Meerut and Powarkheda fall under mega environment (ME)-1 and (ME)-5 respectively, and the soil types of these two locations are deep clay soil and deep loam soil, respectively. At each location, evaluation of WAM panel under both IR and RF conditions minimized the effect of differences in soil types and other environmental conditions on the phenotypic variations at the two locations.

The panel was grown in a simple lattice design with two replications. Each plot, representing an individual genotype, consisted of three 150-cm-long rows with row to row distance of 25 cm. Five irrigations were given under IR condition, while only one irrigation (for sustaining the crop) was given under RF condition (21 days after sowing) to ensure water stress. The details of the experimental locations, sowing dates, meteorological data, and rainfall data are summarized in Supplementary Table 1.

### Phenotypic Evaluation and Data Analysis

Phenotypic data were recorded for the following four traits: (i) days to anthesis (DTA): recorded as the number of days from planting until anthers in 70% of the spike in a plot had extruded, (ii) grain filling duration (GFD); difference between the DTA and days to maturity (DTM) which was number of days from planting until 70% of the spikes in each plot had turned yellow), (iii) grain number per ear (GNPE); average number of grains per ear using five representative ears per plot, and (iv) grain weight per ear (GWPE); average weight (*g*) using five ears per plot.

Statistical analysis for all the four traits was conducted using software SPSS 17.0 (http://www.spss.com) to obtain values of range, mean, standard deviation, coefficient of variation (CV), and analysis of variance (ANOVA). To determine the normal distribution, skewness and kurtosis were also analyzed. Correlations between all pairs of traits were obtained separately for IR and RF conditions.

### Stress Indices

Phenotypic data were used to calculate the following seven indices to estimate the level of drought tolerance: (i) Drought resistance index (DI) = $\left(Ys\times (Ys/Yp\right)/\bar{Y}s$ (Lan, 1998); (ii) Geometric mean productivity (GMP) = √(Ys × Yp) (Fernandez, 1992); (iii) Mean productivity index (MPI) = (*Ys* + *Yp*)/2 (Rosielle and Hamblin, 1981); (iv) Relative drought index (RDI) = $\left(Ys/Yp\right)/\left(\bar{Y}s/\bar{Y}p\right)$ (Fischer and Maurer, 1978); (v) Stress tolerance index (STI) = $\left(Ys\times Yp\right)/\left(\bar{Y}\;p\right)ˆ2\;$(Fernandez, 1992); (vi) Yield index (YI) = $Ys/\left(\bar{Y}s\right)$ (Gavuzzi et al., 1997); (vii) Yield stability index (YSI) = *Ys*/*Yp* (Bouslama and Schapaugh, 1984), where *Ys* is the trait value under drought stress and *Yp* is the value for the same trait under normal condition.

### Genotyping, Population Structure, and LD

SNP data was generated using the genotyping-by-sequencing (GBS) approach developed by DArT Pty. Ltd., Yarralumla, Australia. The details of the methodology are described by Sehgal et al. (2015). Population structure and linkage disequilibrium (LD) information were available from our earlier study (Gahlaut et al., 2019). Briefly, 9627 SNPs with missing data (<30%) and minor allele frequency (MAF) >5% were used for genotyping. These SNPs were randomly distributed across all the 21 wheat chromosomes spanning 5943.1 cM with an average of 17 SNPs per 10 cM genetic distance. Model-based cluster analysis was performed for population structure using Software STRUCTURE version 2.2 (Pritchard et al., 2000) assuming number of subpopulations (*K*) to range from 2 to 20, and burn-in and Markov Chain Monte–Carlo (MCMC) iteration were set to 50,000 and 100,000, respectively. The actual number of subpopulations was determined using the tool “STRUCTURE Harvestor” following delta *K* (Δ*K*) method (Evanno et al., 2005). LD was estimated using software TASSEL v. 5.0 (Bradbury et al., 2007); mean LD values were obtained for the whole genome as well as for individual chromosomes.

### Multi-Locus Genome Wide Association Mapping

SNPs with no more than 30% missing data and >5% minor allele frequency were utilized for GWAS. Principle component analysis (PCA) was conducted using TASSEL v5.0, and first three components were incorporated as a covariate in association test model. Fixed and random model Circulating Probability Unification (FarmCPU), developed by Liu et al. (2016) was used for ML-GWAS. This method is believed to be the most efficient and eliminates confounding issues arising due to population structure, kinship, multiple testing, etc. The method utilizes both Fixed Effect Model (FEM) and a Random Effect Model (REM), iteratively. FEM allows identification of associated markers (MTAs) that are described as pseudo-quantitative trait nucleotides (pseudo-QTNs) and are used as covariates, in REM, which allows identification of QTNs; in this study, QTNs are described as MTAs. Bonferroni-correction was built in within FarmCPU, so that a default *P*-value threshold (0.01) was used to declare significant MTAs. Quantile–quantile (Q–Q) plots generated through FarmCPU were used to examine model fitting (account for population structure). Phenotypic differences between the two alleles of SNPs identified as MTA were tested using “*t*-test” analysis.

### Joint Effect of MTAs and Identification of Contrasting Genotypes

Wherever more than two SNPs were associated with the same trait, joint effects were estimated. This was done through linear regression performed using all desirable SNP alleles for the trait (independent variable) and corresponding trait values of the genotypes that contained more than one desirable SNP alleles (dependent variable). Contrasting genotypes were identified using phenotypic values for all the four traits under IR and RF conditions. For this purpose, average sum of ranks (ASR) of seven indices was calculated for each genotype. In the case of DTA, genotypes with higher ASR were considered superior, while for GFD, GNPE, and GWPE, genotypes with lower ASR were considered desirable.

### Identification of CGs and Expression Analysis

CGs for the associated SNPs were identified by aligning the associated GBS sequences to wheat genome assembly IWGSC refSeq v1.1 available in the Ensemble database (http://www.ensembl.org/info/docs/tools/vep/index.html). The GO annotation (including molecular function and biological process) of each CG was extracted from the IWGSC website (http://www.wheatgenome.org/). Information about expression of CGs was collected using the online tool Genevestigator (Hruz et al., 2008).

## Results

### Phenotypic Variation and Effect of Water Stress on Grain Weight-Related Traits

**Figure 1:**
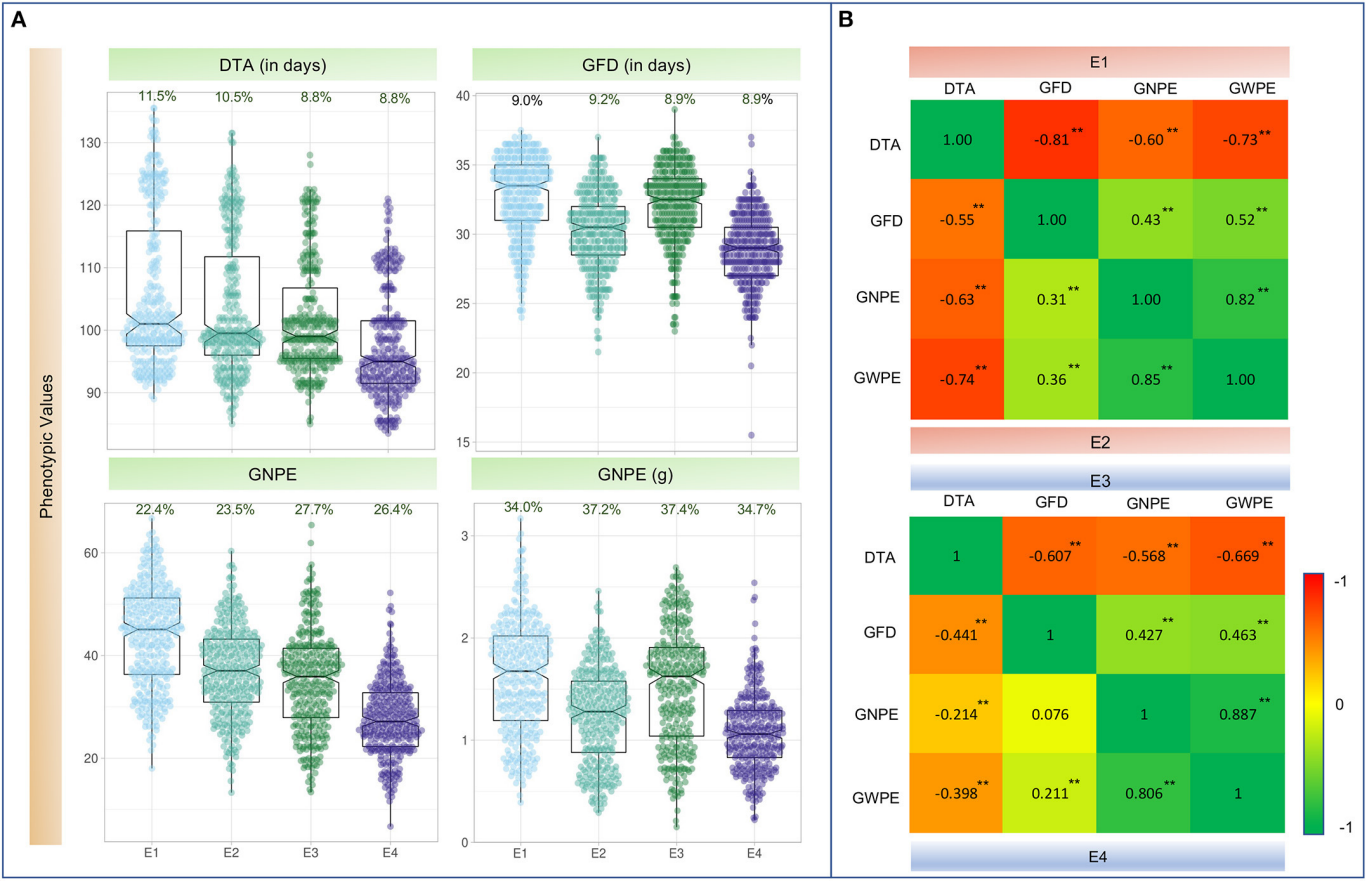
**(A)** Boxplots showing the distribution of values for four traits in four environments. For each trait, its coefficient of variation % (CV%) is displayed on top of each plot. **(B)** Heatmap showing Pearson's correlation coefficients (*r*-values) among four traits. Values above the diagonal on the left are *r*-values in irrigated environments (E1 and E3); values below the diagonal on the right indicate *r*-values in rainfed environments (E2 and E4); **Significant at *P*_0.001; DTA, days to anthesis; DTM, days to maturity; GFD, grain filling duration; GNPE, grain number per ear; GWPE, grain weight per ear. E1, Meerut Irrigated (IR); E2, Meerut rainfed (RF); E3, Powarkheda irrigated (IR); E4, Powarkheda rainfed (RF).

The WAM panel exhibited wide range of phenotypic variation for each of the four traits, under both IR and RF conditions at each of the two locations (Figure 1A; Supplementary Table 2). Skewness and kurtosis were found within the range of normal distribution (i.e., ±2.0) for all the traits under all environmental conditions. The only exception was GFD in E4, where kurtosis was observed with a value of 2.2. It is also apparent that each of the four traits was adversely affected under RF condition at both the locations (Meerut and Powarkheda; Figure 1A; Supplementary Table 2).

ANOVA suggested that genotypes differed for the four traits. The performance of genotypes differed under IR and RF and at the two locations (Meerut and Powarkheda) for each of the traits. Genotype × location interactions were significant for DTA and GNPE, while genotype × environment interactions (IR vs. RF) were significant for DTA and GFD (Table 1). Correlations were largely significant under IR and RF conditions at both the locations, except those between GNPE and GFD under RF at Powarkheda (Figure 1B).

**Table 1:** Analysis of variance (ANOVA) showing mean squares (MS) for four grain weight-related traits.

**Source of variation**	**df**	**DTA**	**GFD**	**GNPE**	**GWPE**
Location	1	8,610^**^	307^**^	25,493^**^	5^**^
Treatment	1	3,897^**^	2,512^**^	15,471^**^	54^**^
Genotypes	313	354^**^	19^**^	203^**^	1^**^
Genotypes × Location	313	43^**^	3	43^*^	0.1
Genotypes × Treatment	313	304^**^	36^**^	23.518	0.3
Error	314	13	4	36	0.1

*DTA, days to anthesis; DTM, days to maturity; GFD, grain filling duration; GNPE, grain number per ear; GWPE, grain weight per ear; df, degree of freedom.*

*,** *Indicate 0.05 and 0.01 levels of significance, respectively*.

### Population Structure and LD

The details about population structure and LD are available in our earlier study (Gahlaut et al., 2019). In brief, three sub-populations involving 157 genotypes were recognized; the remaining 163 genotypes were admixed. The number of genotypes was 57 in sub-population I, 85 in sub-population II and 15 in sub-population III. Genome-wide LD decay was observed at 3 cM with a range of 2–20 cM in different genomic regions.

### Marker-Trait Association

**Figure 2:**
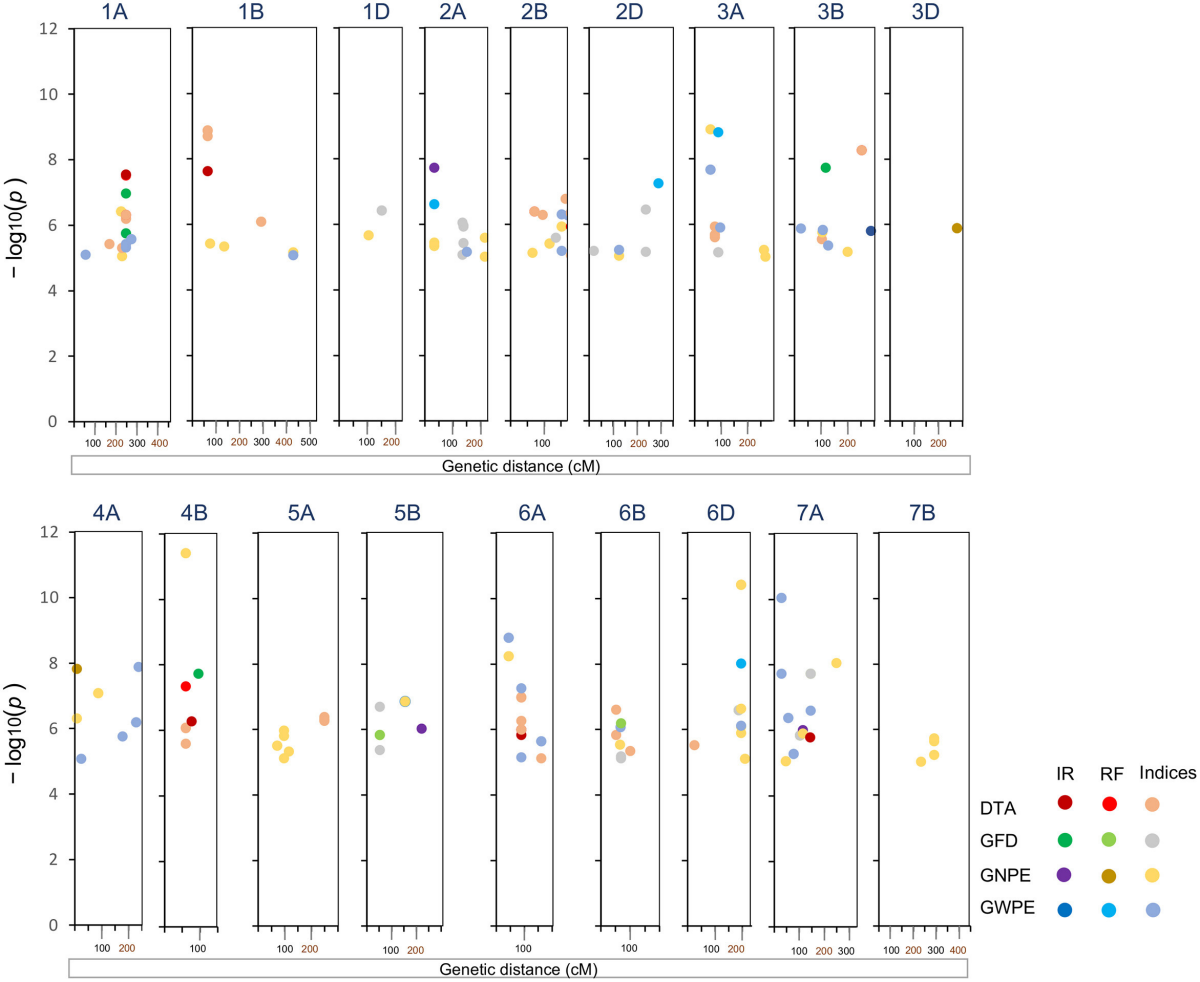
Significant markers trait associations (MTAs) for four traits and seven indices identified on 18 chromosomes following ML-GWAS. DTA, days to anthesis; DTM, days to maturity; GFD, grain filling duration; GNPE, grain number per ear; GWPE, grain weight per ear. IR, Irrigated; RF, rainfed.

A total of 30 high confidence MTAs were detected for the four traits under one or more environmental conditions. These MTAs involved 27 SNPs distributed over 15 chromosomes (excluding 1D, 4D, 5A, 5D, 7B, and 7D) (Table 2; Figure 2). Manhattan and Q–Q plots showing appropriate model fitting for ML-GWAS are shown in Supplementary Figure 1. Only three SNPs (SNP_404, SNP_1555, and SNP_8047) had association with more than one trait. For individual trait, a maximum of nine MTAs were available for DTA while a minimum of six MTAs were available for GWPE. For DTA, out of nine MTAs, four SNPs (SNP_647, SNP_5369, SNP_7068, and SNP_8390) were uniquely identified under IR condition, four other SNPs (SNP_2283, SNP_2860, SNP_448, and SNP_5304) under RF condition, and one MTA (SNP_404) was identified under both IR and RF conditions. The effect size of these 9 MTAs ranged from 2.2 to 5.2. The effect size of alternate alleles of SNPs associated with DTA was significant in different environments (Supplementary Figure 2). Seven SNPs were associated with GFD, eight with GNPE, and six with GWPE. Effect sizes for associated SNPs were also estimated for individual traits (Table 2).

**Table 2:** List of MTAs for four grain weight-related traits.

**Trait/SNP_ID (SNP alleles)**	**Chr**.	**Position: cM/bp**	**-log (p)**	**SNP effect**	**Desirable allele (Freq.)**	**Environment**
**Days to anthesis (DTA)**						
SNP_404 (G/T)	1A	247.9/ 519892942	6.3–7.5	2.56–4.06	G (0.78)	E1, E2, E4
SNP_647 (G/T)	1B	64.8/525928165	7.6	5.17	G (0.90)	E1
SNP_2283 (T/A)	2B	71.4/57887257	6.4	−1.5	A (0.53)	E4
SNP_2860 (C/G)	2B	179.5/772063522	5.9	3.34	C (0.92)	E2
SNP_4482 (C/A)	3B	253.7/808103956	8.3	2.25	C (0.28)	E4
SNP_5304 (T/G)	4B	60.1/39758292	7.3	−3.87	G (0.91)	E2
SNP_5369 (T/C)	4B	76.3/562867086	6.3	−2.63	C (0.94)	E3
SNP_7068 (C/T)	6A	88.9/116943461	5.8–6.0	2.29–2.97	C (0.78)	E1, E3
SNP_8390 (A/G)	7A	144.2/129879691	5.8	2.51	A (0.86)	E3
**Grain filling duration (GFD)**						
SNP_404 (G/T)	1A	247.9/ 519892942	7	−1.2	G (0.78)	E1
SNP_4163 (G/A)	2B	117.6/506732534	7.7	0.72	A (0.51)	E1
SNP_5442 (C/T)	4B	95.4/620061711	7.7	−1.48	C (0.96)	E3
SNP_6185 (A/G)	5B	51.8/87491998	5.8	0.57	G (0.55)	E2
SNP_8018 (C/T)	6D	186.1/456804609	6.6	0.79	T (0.73)	E2
SNP_8312 (C/G)	7A	102.1/94170034	5.8	0.81	G (0.13)	E4
SNP_8407 (A/G)	7A	145.4/159707863	7.7	−0.87	A (0.80)	E2
**Grain number per ear (GNPE)**						
SNP_359 (G/T)	1A	224.7/507956069	6.4	1.41	T (0.53)	E2
SNP_1555 (G/A)	2A	34.0/5654868	7.7	3.39	A (0.13)	E3
SNP_4743 (C/T)	3D	275.4/602203097	5.9	−1.96	C (0.84)	E4
SNP_4793 (G/T)	4A	7.2/13655378	7.8	3.10	T (0.14)	E1
SNP_6510 (A/C)	5B	153.6/565154441	6.9	−1.56	A (0.55)	E2
SNP_6636 (C/G)	5B	220.8/656080718	6	−1.51	C (0.53)	E3
SNP_8047 (A/T)	6D	196.2/467856107	10.4	−2.84	A (0.68)	E2
SNP_8336 (T/C)	7A	114.9/85601215	6	1.87	C (0.58)	E3
**Grain weight per ear (GWPE)**						
SNP_1555 (G/A)	2A	34.0/5654868	6.6	0.17	A (0.13)	E3
SNP_3180 (T/G)	2D	289.6/639092898	7.2	−0.14	T (0.11)	E3
SNP_3501 (T/C)	3A	89.8/510721706	8.8	0.15	C (0.41)	E3
SNP_4548 (T/C)	3B	287.8/819037501	5.8	−0.08	T (0.67)	E2
SNP_7747 (C/A)	6B	69.0/649814798	6.2	−0.11	C (0.65)	E2
SNP_8047 (A/T)	6D	196.2/467856107	8	−0.17	A (0.68)	E1

*Showing associated SNPs SNP allele, SNP effects, desirable alleles and their frequencies. E1, Meerut irrigated; E2, Meerut rainfed; E3, Powarkheda irrigated; E4, Powarkheda rainfed*.

Based on the effect size, desirable alleles of associated SNPs were also selected (Table 2). Among the four traits, positive selection appeared desirable for GFD, GNPE, and GWPE, and negative selection for DTA. Higher absolute value of SNP effect size showed higher contribution of SNP on the phenotype. Frequency of desirable alleles ranged from 0.10 to 0.94. Two SNPs exhibited pleiotropic effect. SNP_404 was associated with DTA as well as GFD in E1. Similarly, SNP_1555 was associated with GNPE and GWPE in E3.

### Joint Effect of Significant SNPs on Associated Phenotypes

**Figure 3:**
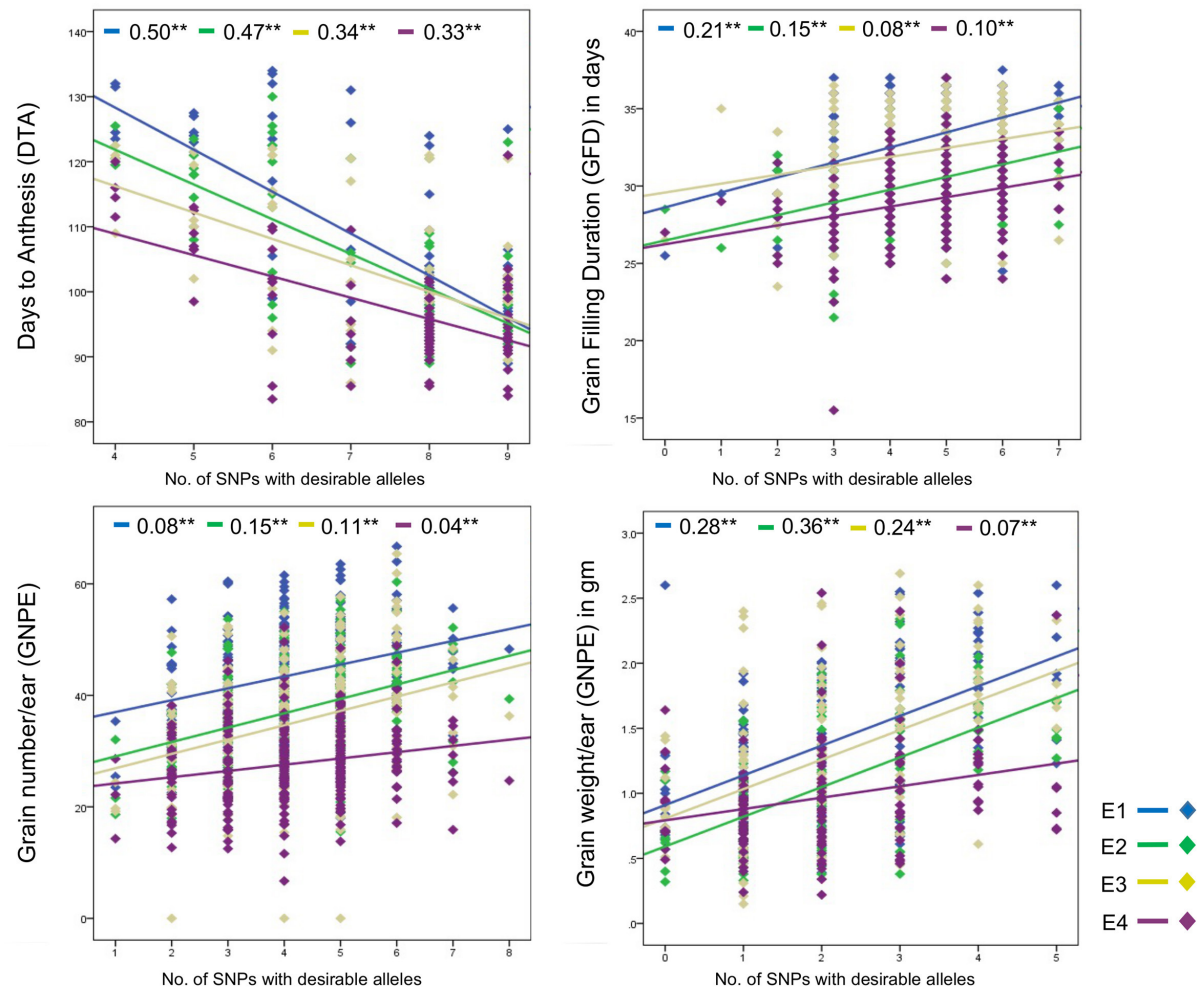
Regression plots showing joint effect of multiple SNPs associated with the same trait. **Shows significant (*p* < 0.01) difference and values written before star are regression coefficient.

Joint effect of desirable alleles of multiple associated SNPs was determined using linear regression. For DTA, nine SNPs were each associated with the trait in one or more environments. An increase in the number of desirable SNP alleles from four to nine (but not less than four) led to a significant decrease in DTA (Figure 3). Interestingly, significant joint effect of nine SNPs on DTA was observed in all the four environments; however, strength of regression varied across environments and ranged from 0.33 (E4) to 0.50 (E1).

Significant joint effects of associated SNPs were also observed for GFD, GNPE, and GWPE. Trait values for each of these three traits increased with an increase in the number of desirable alleles in all the four environments (Figure 3). The regression coefficients ranged from 0.08–0.21 for GFD, 0.08–0.15 for GNPE, and 0.07–0.36 for GWPE.

### Analyses of Trait Indices

Seven different stress-related indices were obtained for each of the four traits to better assess the genetics of drought tolerance at both locations (total of 28 index traits); this allowed the identification of 153 MTAs involving 85 SNPs (Supplementary Table 3). Manhattan and Q–Q plots showing appropriate model fitting for ML-GWAS tests are shown in Supplementary Figure 3. These SNPs were distributed over 18 of the 21 wheat chromosomes (except 4D, 5D, and 7D). A comparison of MTAs for 28 indices, including seven indices for each of the four main traits allowed the identification of 19 common SNPs associated with response to water stress (Table 2; Supplementary Table 3). As many as 19 SNPs for DTAs, 13 SNPs for GFD, 34 SNPs for GNPE and 28 SNPs for GWPE showed significant association with one or more indices (Supplementary Table 3).

### Contrasting Genotypes for Molecular Breeding Programs

Using ASR values of indices, two contrasting genotypes were selected, which included the superior genotype TX181, and the inferior genotype TX67 (Table 3). TX181 performed better under RF condition for all the four traits (Table 3). These two genotypes can be used for further studies involving crosses generating segregating populations for fine-mapping of QTLs leading to cloning.

**Table 3:** The two selected genotypes (TX181 and TX67) showing variation in the mean values of four traits under irrigated (IR) and rainfed RF environments, the % decline of trait value under RF condition, alleles of significant SNPs.

**Contrasting genotype**	**Trait**	**IR**	**RF**	**% dif**.	**Alleles of significantly associated SNPs**
TX181	DTA	96.25	92.25	4.16	**SNP_404-GG, SNP_647-GG, SNP_2283-AA, SNP_2860-CC, SNP_4482-CC, SNP_5304-GG, SNP_5369-CC, SNP_7068-CC**, ***SNP_8390-NN***
	GFD	34.25	32.50	5.11	**SNP_404-GG, SNP_4163-AA, SNP_5442-CCSNP_6185-GG, SNP_8018-TT, SNP_8312-CC, SNP_8407-AA**
	GNPE	52.23	41.58	20.39	**SNP_359-TT, SNP_1555-GG, SNP_4743-CC, SNP_4793-GG, SNP_6510-CC, SNP_6636-CCSNP_8047-AASNP_8336-CC**
	GWPE	1.91	1.58	17.28	**SNP_1555-GG**, ***SNP_3180-NN***, **SNP_3501-CC, SNP_4548-TT, SNP_7747-CC, SNP_8047-AA**
TX67	DTA	120.50	119.00	1.24	**SNP_404-GG, SNP_647-TT, SNP_2283-TT, SNP_2860-GG, SNP_4482-CC, SNP_5304-TT**, ***SNP_5369-NN***, **SNP_7068-CC, SNP_8390-GG**
	GFD	28.25	25.25	10.62	**SNP_404-GG, SNP_4163-AA**, SNP_5442-TT, SNP_6185-AA, *SNP_8018-NN, SNP_8312-NN*, SNP_8407-GG
	GNPE	28.23	14.85	47.40	**SNP_359-TT, SNP_1555-GG, SNP_4743-TT, SNP_4793-GG, SNP_6510-AA, SNP_6636-CC**, ***SNP_8047-NN***, **SNP_8336-TT**
	GWPE	0.62	0.27	56.45	**SNP_1555-GG, SNP_3180-TT, SNP_3501-TT, SNP_4548-CC, SNP_7747-AA, SNP_8047-NN**

*NN represents missing genotypic data. Desirable alleles of SNPs are in bold font. % dif., % difference*.

The pattern of decline in trait values in RF condition was also examined to assess the sensitivity of these two genotypes to water stress. For this purpose, per cent decline in trait value under RF was examined. Interestingly, in case of TX181, the reduction in trait value under RF condition was relatively low [GFD, 5.11%; GNPE, 20.39%; and GWPE, 17.28%], when compared with those for TX67 [GFD, 10.62%; GNPE, 47.45%, GWPE, 56.45%]. In case of DTA, where lower value is desirable, larger decline was observed in TX181 (4.16%) relative to TX67 (1.24%) under RF condition. However, the DTA in TX67 was about one month longer than TX181. These observations revealed that TX181 is less sensitive (more tolerant) and TX67 is more sensitive under water stress conditions. We may therefore conclude that these varieties differ widely not only for absolute trait values, but more importantly, for tolerance to drought stress.

The above contrasting genotypes were also examined for the presence of desirable alleles of significant SNPs, assuming that desirable alleles for all SNPs are unlikely to be concentrated in TX181; similarly, undesirable alleles for all SNPs cannot be present in TX67. For some of the SNPs, the two genotypes may not differ. Interestingly, out of 27 significantly associated SNPs for four traits, desirable alleles of 24 SNPs were present in TX181. For one SNP, an undesirable allele was found and for two SNPs genotypic data were missing in TX181. In contrast, in TX67, undesirable alleles were present for 14 SNPs, desirables for 9 SNPs, and for the remaining four SNPs, genotypic data were missing (Table 3).

### Candidate Genes (CGs) Co-Localized With Associated SNPs

The 27 SNPs involved in 30 MTAs (as mentioned above) were used to identify candidate genes (CGs). As many as 10 of the 27 SNPs were co-located within protein-coding genes and were therefore treated as putative CGs. Details of CGs and their corresponding annotation information are provided in Table 4. Eight of these 10 CGs represent those MTAs that were detected either in IR or RF environments; two MTAs were detected in both the environments. GO annotations of the CGs showed their involvement in protein binding, innate immune response, microtubule cytoskeleton organization, protein self-association, protein phosphorylation and protein folding (Table 4).

**Table 4:** Description of candidate genes (CGs) associated with significant MTAs and, GO annotations and details of putative proteins identified from Ensembl wheat.

**Associated SNP**	**Trait _Environment**	**Overlapping gene^#^**	**SNP position (location in gene)**	**GO Annotation**			**Gene description**
				**Biological process (ID)**	**Molecular function (ID)**	**Cellular component (ID)**	
SNP_404	DTA_E1, DTA_E2, DTA_E4, GFD_E1	TraesCS1A02 G331000	519892942 (Intron1)	N/A	N/A	N/A	RNA helicase
SNP_3180	GWPE_E3	TraesCS2D02 G574400	639092898 (3'UTR)	Innate immune response-activating signal transduction (GO:0002758)	ADP binding (GO:0043531)	Cytoplasm (GO:0005737)	Disease resistance protein RGA5-like
SNP_3501	GWPE_E3	TraesCS3A02 G282200	510721706 (3'UTR)	N/A	Protein binding (GO:0005515)	N/A	F-box family protein
SNP_4548	GWPE_E2	TraesCS3B02 G596100	819037501 (Exon1)	N/A	N/A	N/A	F-box family protein
SNP_4793	GNPE_E1	TraesCS4A02 G019800	13655378 (Intron3)	N/A	N/A	N/A	DUF1997 family protein
SNP_5304	DTA_E2	TraesCS4B02 G051200	39758292 (Intron3)	Microtubule cytoskeleton organization (GO:0000226)	Microtubule binding (GO:0008017)	Microtubule (GO:0005874)	Microtubule-associated protein (MAP65/ASE1 family)
SNP_5442	GFD_E3	TraesCS4B02 G329500	620061711 (3'UTR)	Defense response (GO:0006952)	Protein self-association (GO:0043621)	Plasma membrane (GO:0005886)	Peptidoglycan-binding lysin motif-containing protein
SNP_8047	GNPE_E2, GWPE_E1	TraesCS6D02 G394600	467856107 (Exon1)	Protein phosphorylation (GO:0006468)	Protein serine/threonine kinase activity (GO:0004672)	Plasma membrane (GO:0005886)	Wall-associated receptor kinase (WAKs) protein
SNP_8336	GNPE_E3	TraesCS7A02 G133300	85601215 (Intron3)	N/A	N/A	N/A	HNH endonuclease
SNP_8390	DTA_E3	TraesCS7A02 G176600	129879691 (Intron18)	Protein folding (GO:0006457)	ATP binding (GO:0005524)	Chloroplast thylakoid membrane (GO:0009535)	Chaperone protein dnaJ 1/HSP40

# *Pfam database IDs*.

Gene expression analysis for the 10 CGs is shown in Supplementary Figures 4–6. This analysis provides further support to their potential involvement in the trait phenotype in different wheat developmental stages and tissues under drought/water stress conditions. The results showed a wide range of expression. For instance, following four CGs had relatively higher expression in all wheat tissues/organ: TraesCS1A02G331000, TraesCS4B02G329500, TraesCS7A02G133300, and TraesCS7A02G176600. Some CGs had tissue-specific expression; for example, TraesCS6D02G394600 expresses in leaf tissues, TraesCS4B02G051200 in root tissues, Traes3B02G596100 in rachis while, TraesCS2D02G574400 in the shoots (Supplementary Figure 4). The CGs also showed varied expression during the different wheat development stages. Interestingly, most CGs had high expression during anthesis to ripening stages except one (Traes3B02G596100), demonstrating their possible role in regulating wheat yield (Supplementary Figure 5).

**Figure 4:**
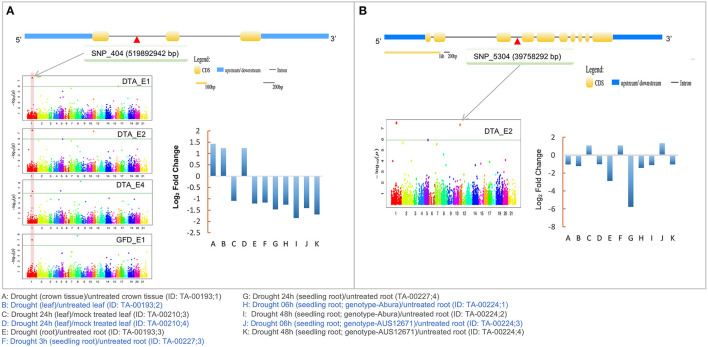
Details of two candidate genes **(A)** TraesCS1A02G331000 (RNA helicase) **(B)** TraesCS4B02G051200 (Microtubule-associated protein; MAP65) associated with SNPs identified in the present study. Structure of the CGs and expression profile of CGs during drought stress is also shown. The candidate gene position in Manhattan plot is shown by gray arrows. Physical location of the SNP on gene shown as red triangle.

Under drought/water stress condition, only five of the 10 CGs showed differential expression (≥2-fold), either down-regulated or up-regulated (Supplementary Figure 6). For instance, TraesCS7A02G133300 (up to ~2-fold), TraesCS4B02G051200 (up to ~6-fold), and TraesCS1A02G331000 (up to ~2-fold) were down-regulated. Similarly, TraesCS4B02G329500 (up to ~2-fold) and TraesCS4A02G019800 (up to ~4-fold) were up-regulated during drought stress. Interestingly, out of these five, one CG (TraesCS4B02G051200) that encodes a microtubule-associated protein (MAP65), was associated with MTAs that were identified only in RF environment. Another CG (TraesCS1A02G331000) that encodes RNA helicase protein, belonged to MTAs that were identified in both IR and RF environments (Figure 4).

## Discussion

In the most major crops including wheat, drought tolerance is a complex polygenic trait involving a large number of minor quantitative trait loci (QTLs; Bernardo, 2008; Gupta et al., 2017) and only a few major QTLs (Bernardo, 2008; Gupta et al., 2012, 2017). A large number of traits (>40) have been utilized to estimate drought tolerance (our unpublished results). In a recent study on meta QTL analysis for drought tolerance, as many as 340 QTLs identified through at least 14 interval mapping studies have been utilized (Kumar et al., 2020). As many as 750 MTAs, were also identified using GWAS (Gupta et al., 2017, 2020; Kumar et al., 2020; Singh et al., 2021). It also seems that the QTLs and MTAs identified so far do not represent the entire genetic variation for a multitude of traits that are involved in providing drought tolerance. It is also known that despite this enormous literature, very few QTLs have been utilized in molecular breeding and pyramiding, and that none of them cloned so far in wheat (Ray et al., 2013; Merchuk-Ovnat et al., 2016; Gautam et al., 2021). Therefore, one would expect that every new study leads to identification of some novel QTLs and MTAs. Perhaps, metaQTLs and ortho-metaQTLs identified recently and to be identified in future (our unpublished results), may lead to a more fruitful utilization of molecular markers for MAS leading to the development of drought tolerant wheat cultivars.

In the present study, 30 MTAs were detected and compared with earlier studies. Five MTAs (all identified under RF conditions) were co-localized with QTL/MTAs identified earlier using either linkage mapping or LD-based GWAS (Table 5). We assume that the remaining 25 MTAs are novel. The five co-localized MTAs listed in Table 5, include the following: (i) SNP for DTA on 4B co-localized with a QTL for DTH and GY (*QDH.ndsu.4B*; Rabbi et al., 2021). (ii) SNP for DTA on 2B co-localized with a SNP for DTH (Gahlaut et al., 2019). (iii) SNP for GFD on 5B, co-localized with a QTL (QGfd.ccsu-5B; 40.6-53.4 cM) for GFD (Gahlaut et al., 2017). (iv) SNP for GFD on 7A (145.43 cM) co-localized with a marker (wsnp_CAP7_c1321_664478~IACX7848) associated with DTA and GY (Qaseem et al., 2018). (v) SNP for GWPE on 6B, co-localized with MTAs/QTL for thousand grain weight (TGW) and grain yield, identified in three earlier studies (Mathews et al., 2008; Ahmed et al., 2020; Rabbi et al., 2021). The markers identified in these three earlier studies include a SSR marker gwm132 on 6B (67.10 cM) associated with GY under DS (Mathews et al., 2008). The other two co-localized genomic region at 64.82 cM (*QTKW.ndsu.6B*) and at 67.24 cM (BS00063801_51) were associated with TGW under DS (Ahmed et al., 2020; Rabbi et al., 2021). These QTLs/MTAs can be utilized for MAS with higher level of confidence. The above genomic regions associated with DTA, GFD, GNPE, and GWPE under drought stress identified in the present study could also be exploited for fine mapping.

**Table 5:** Comparison of MTAs identified in present study with QTL/MTAs reported in earlier studies.

**MTAs (present study)**			**QTL/MTA previously reported**			**References**
**Trait/SNP**	**Chr.; Pos. (cM)**	**Env**.	**QTL/Marker Associated**	**Chr.; Pos. (cM)**	**Trait**	
**Days to anthesis (DTA)**						
SNP_2860	2B; 179.52	E2	SNP_2860	2B; 179.52	DTH under DS	Gahlaut et al., 2019
SNP_5304	4B; 60.12	E2	*QDH.ndsu.4B*	4B; 64.03	DTH, GY under DS	Rabbi et al., 2021
**Grain filling duration (GFD)**						
SNP_6185	5B; 51.85	E2	*QGfd.ccsu-5B*	5B; 40.6-53.4	GFD under DS	Gahlaut et al., 2017
SNP_8407	7A; 145.43	E2	wsnp_CAP7_c13 21_664478~IACX7848	7A; 148.4-160	DTA, GY under DS	Qaseem et al., 2018
**Grain weight per ear (GWPE)**						
SNP_7747	6B; 69.05	E2	gwm132	6B; 67.10	GY under DS	Mathews et al., 2008
			BS00063801_51	6B; 67.24	TGW under DS	Ahmed et al., 2020
			*QTKW.ndsu.6B*	6B; 64.82	TGW under DS	Rabbi et al., 2021

*Chr., chromosome; Pos., position; Env., environment; DTA, days to anthesis; DTH, days to heading; GY, grain yield; TGW, thousand grain weight; DS, drought stress*.

The effect size of individual associated SNPs on an individual trait and the association of same SNP with more than one trait also deserve attention. For 25 of 30 MTAs, the proportion of genotypes with desirable allele was higher relative to that of undesirable effect. This could be due to unconscious selection for these desirable alleles during wheat breeding, and those may be utilized in future breeding as well. More important are the SNPs, which have desirable alleles in just a few genotypes, which therefore deserve priority in future breeding. For instance, SNP_1555 located on chromosome 2A that was associated with GNPE having highest SNP effect size (3.39) had a frequency of desirable allele 0.13; additionally, this SNP was also associated with GWPE and showed a similar pattern. This SNP, therefore, appears promising for future wheat breeding efforts.

Interestingly, some SNPs also showed pleiotropic effect, each showing association with two correlated traits. However, no SNP was available to be associated with more than two traits. The two pleiotropic SNPs included, SNP_404 (DTA and GFD) and SNP_1555 (GNPE and GWPE) and deserve further attention in future studies.

The relative merit of MTAs for the seven indices relative to those for individual traits also deserves attention, since indices have been designed to estimate tolerance to drought. SNPs associated with more than one index traits appear to be relatively important since they provide more comprehensive information about the response of genomic regions toward drought stress. Based on seven different drought stress-related indices, we also identified contrasting genotypes for response to drought (Table 3). These contrasting genotypes may be used for fine mapping and to develop improved wheat lines *via* molecular breeding.

Significant joint effect of multiple associated SNPs was observed where genotypes with desirable alleles for many more associated SNPs showed superior phenotype than those having desirable alleles for fewer SNPs (Figure 3); the trait value can be substantially improved through pyramiding of multiple significant SNPs. Sometimes pyramiding of a large number of SNPs becomes problematic due to the requirement of a larger population. In such cases, marker-assisted recurrent selection (MARS) may be followed.

Among the 10 CGs identified using MTAs, two CGs seem to be involved in response to drought stress and, therefore, deserve special attention. The first of these two genes, namely TraesCS4B02G051200 encodes a microtubule-associated protein (MAP65) and the second gene, namely TraesCS1A02G331000 encodes RNA helicase protein. Both these proteins respond to drought stress and therefore their expression level may be used for measuring the level of drought stress. However, TraesCS4B02G051200 that was associated with DTA and was detected using MTA only in the RF environment, while TraesCS1A02G331000 was associated with MTAs detected under both IR and RF conditions. The proteins of MAP65 family are known to be involved in the polymerization of the microtubules (Hamada, 2014) and are indirectly involved in regulating growth and response to abiotic stresses including drought in plants (Zhang et al., 2012; Bhaskara et al., 2017). In our study, the expression of TraesCS4B02G051200 (wheat MAP65) decreased up to ~6-fold after drought stress (Figure 4B). This means that this gene can be utilized as an indicator of drought stress, both in wheat breeding and in strategic research.

RNA helicase proteins are multifunctional and are involved in responses to both biotic and abiotic stresses in plants (Pandey et al., 2020). For instance, RNA helicase belonging to Arabidopsis RH8 DEAD-box regulates the ABA-signaling pathway by interacting with 2C protein phosphatase (PP2CA), which also plays a vital role in drought tolerance (Baek et al., 2018). Significant variation in the expression of wheat RNA helicase during drought stress was also observed in the present study (Figure 4A), suggesting that this gene may also be used for the estimation of drought stress and for improving drought tolerance. Therefore, we hypothesized that these two CGs together may provide drought resilience in wheat. Further investigation involving functional analyses of these two genes may also help in understanding the molecular mechanism of abiotic stress tolerance in crops.

## Data Availability Statement

The original contributions presented in the study are included in the article/Supplementary Material, further inquiries can be directed to the corresponding author/s.

## Author Contributions

PG and HB: conceptualization. VG and VJ: methodology and formal analysis. VJ: data curation. VG, VJ, and AJ: writing-original draft preparation. PG, HB, VJ, and VG: writing-review and editing. PG, HB, and AJ: supervision. All authors have read and agreed to the published version of the manuscript.

## Conflict of Interest

The authors declare that the research was conducted in the absence of any commercial or financial relationships that could be construed as a potential conflict of interest.

## Publisher's Note

All claims expressed in this article are solely those of the authors and do not necessarily represent those of their affiliated organizations, or those of the publisher, the editors and the reviewers. Any product that may be evaluated in this article, or claim that may be made by its manufacturer, is not guaranteed or endorsed by the publisher.
