# Supplementary material for: Multi-Locus GWAS for Grain Weight-Related Traits Under Rain-Fed Conditions in Common Wheat (Triticum aestivum L.)
Source: Front Plant Sci. 2021 Oct 21;12:758631. doi: 10.3389/fpls.2021.758631 (PMC8568012; doi:10.3389/fpls.2021.758631)
Supplement: Supplementary file 1 [file Data_Sheet_1.PDF]

Supplementary Material

Supplementary Figures

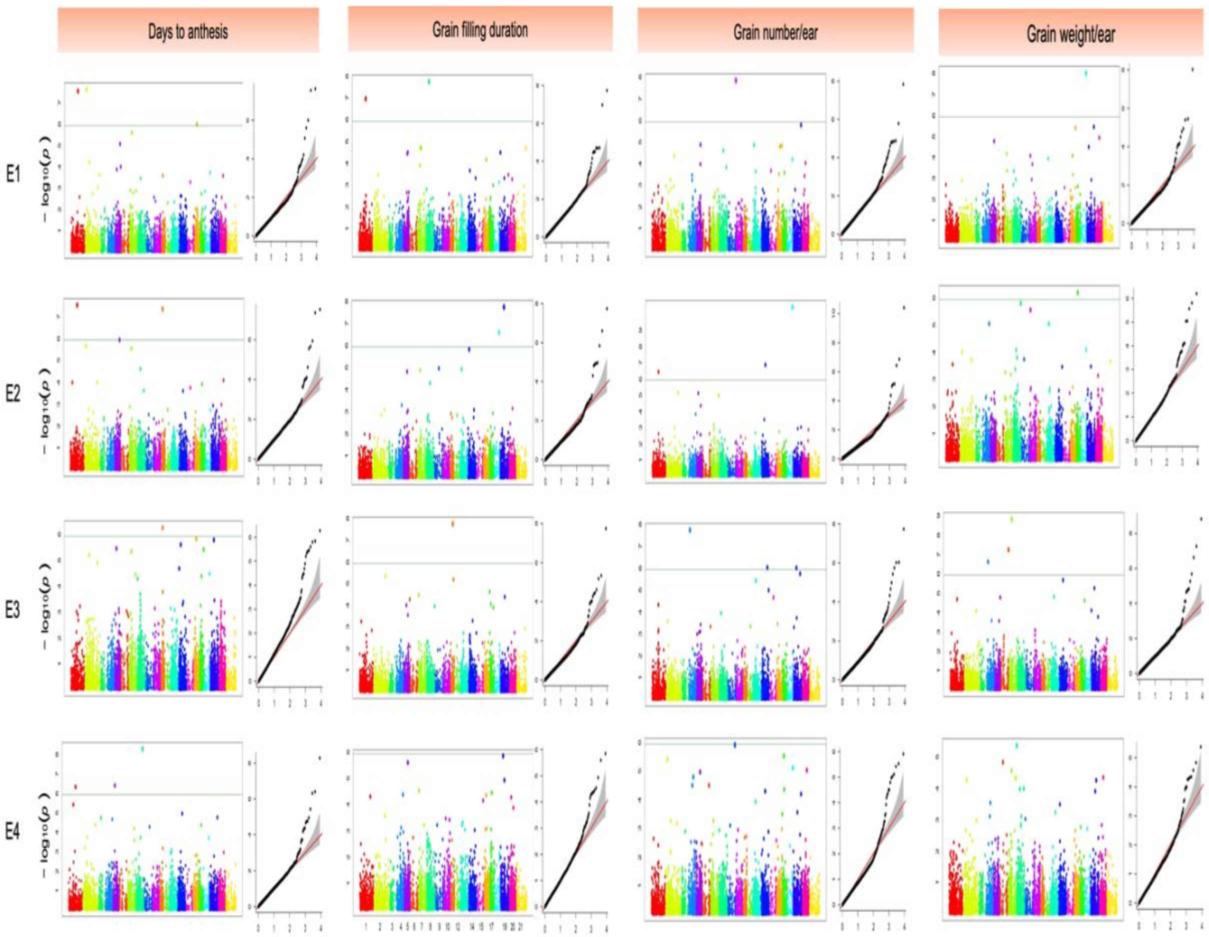

Supplementary Figure S1. Manhattan plots and quantile-quantile (Q-Q) plots of the GWAS results for days to heading (DTA), grain filling duration (GFD), grain number per ear (GNPE), grain weight per ear (GWPE) in four environments (E1, Meerut Irrigated; E2, Meerut rainfed; E3, Powarkheda irrigated; E4, Powarkheda rainfed). Significant MTA threshold [– log 10 (p) <10 $^{-06}$] are represented by green lines.

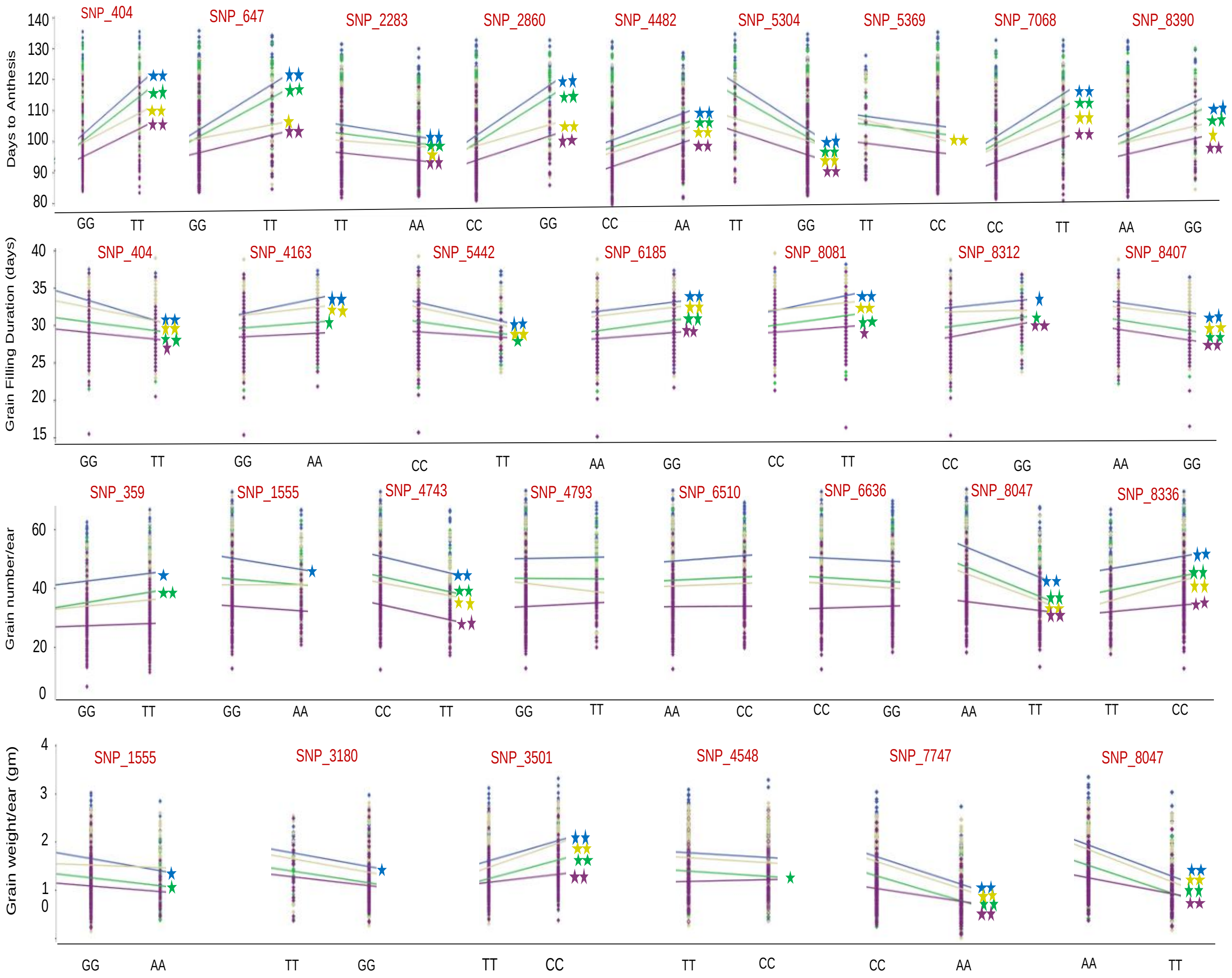

Supplementary Figure S2. Regression plots showing the trait variation with two contrasting alleles of the associated SNPs in four different environments. * significant at 0.05 and ** significant at 0.01 levels.

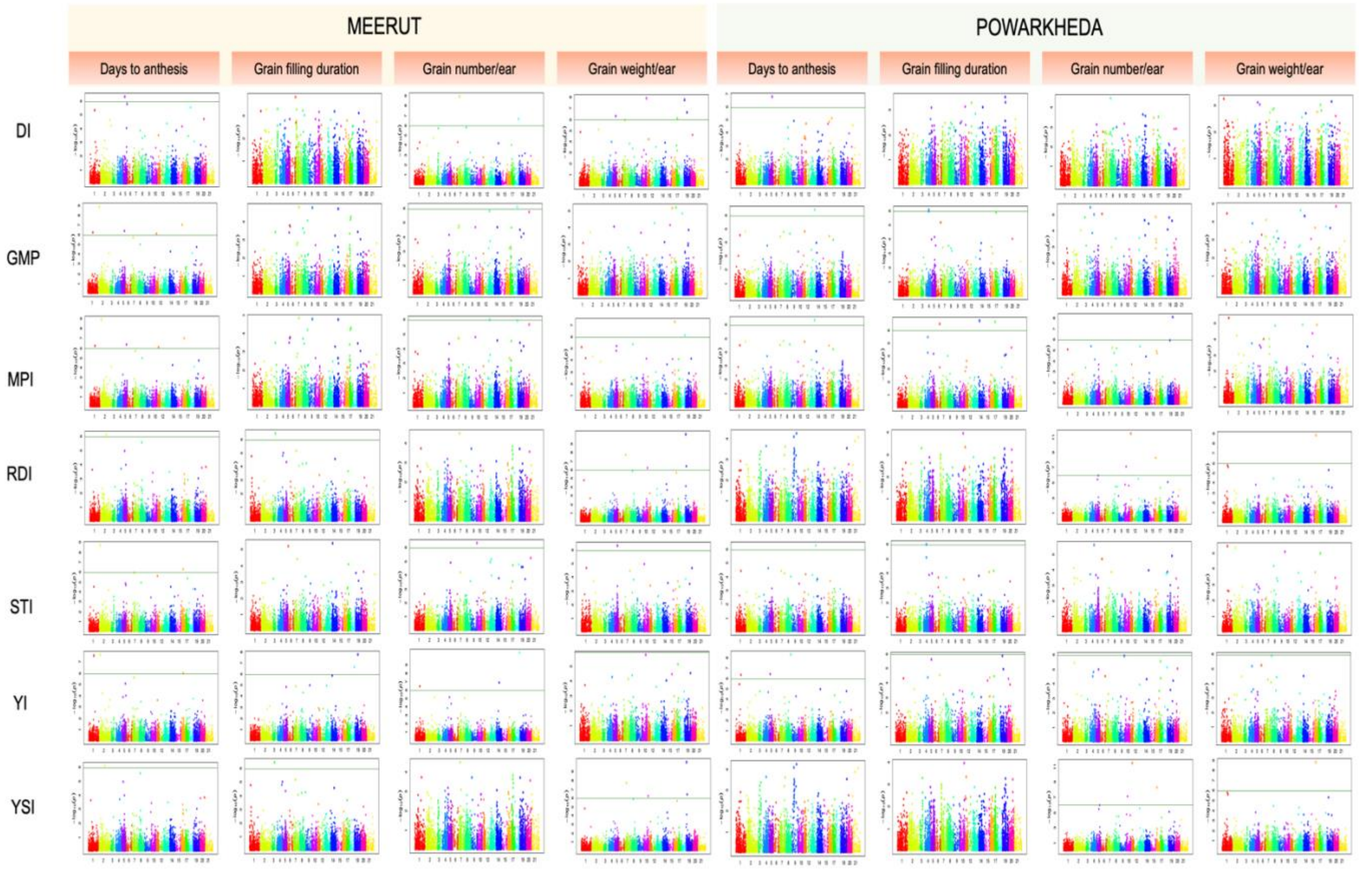

Supplementary Figure S3. Manhattan plots and quantile-quantile (Q-Q) plots for seven different stress related indices for each of the four traits in two locations (Meerut and Powarkheda). Significant MTA threshold [– log 10 (p) <10 $^{-06}$] are represented by green lines. DI, drought resistance index; GMP, geometric mean productivity; MPI, mean productivity index; RDI, relative drought index; STI, stress tolerance index; YI, yield index; YSI, yield stability index.

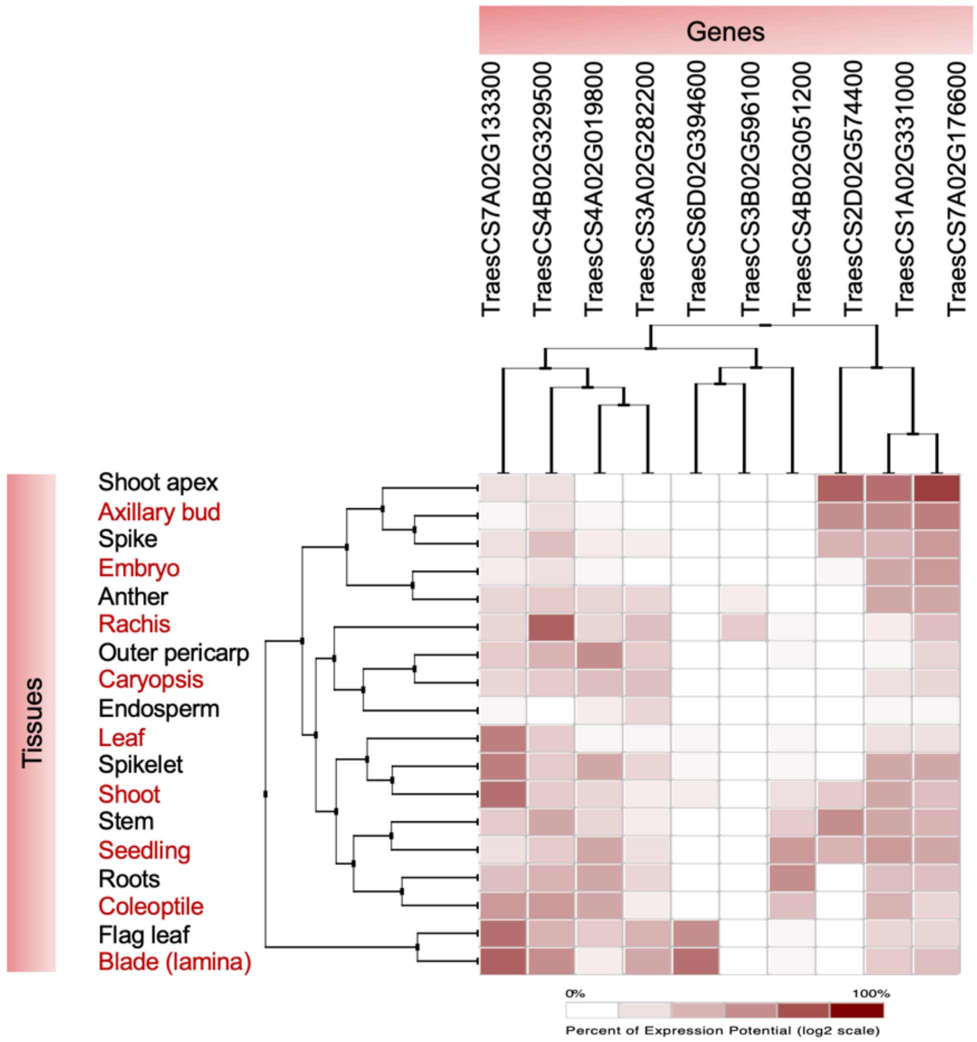

Supplementary Figure S4. Heat map showing the tissue-specific expression patterns of 10 candidate genes (CGs) in different wheat tissues. Colors represent the intensity of the expression (percentage of expression potential), from white (0%) to dark brown (100%).

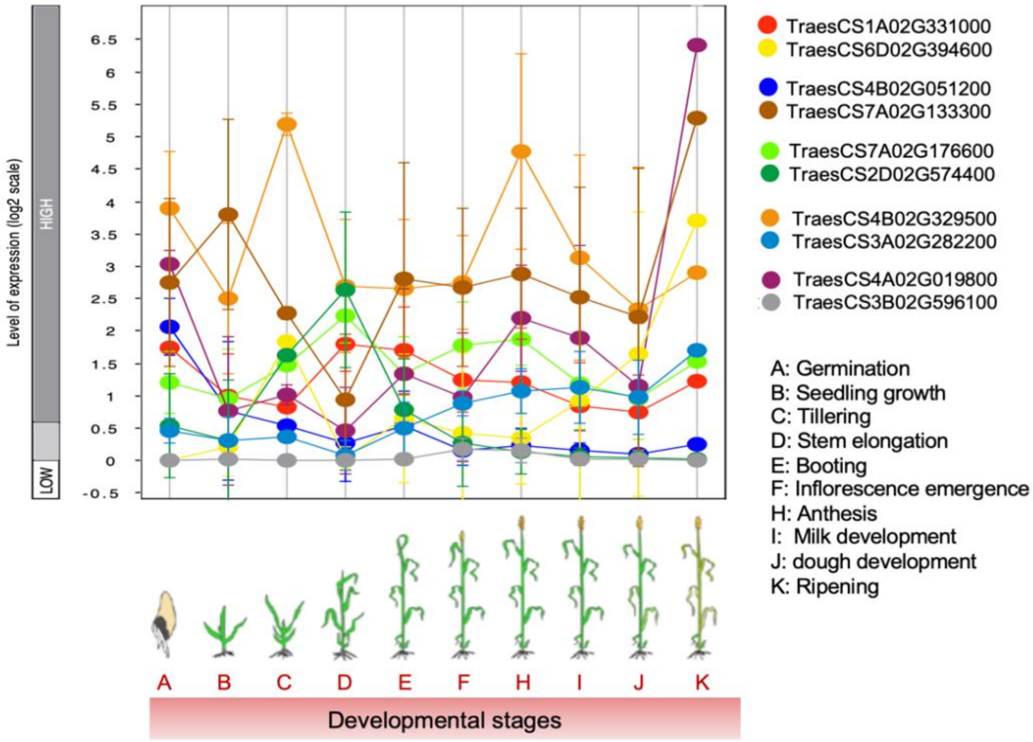

Supplementary Figure S5. Heat map showing the developmental stage-specific expression patterns of 10 candidate genes (CGs) in wheat.

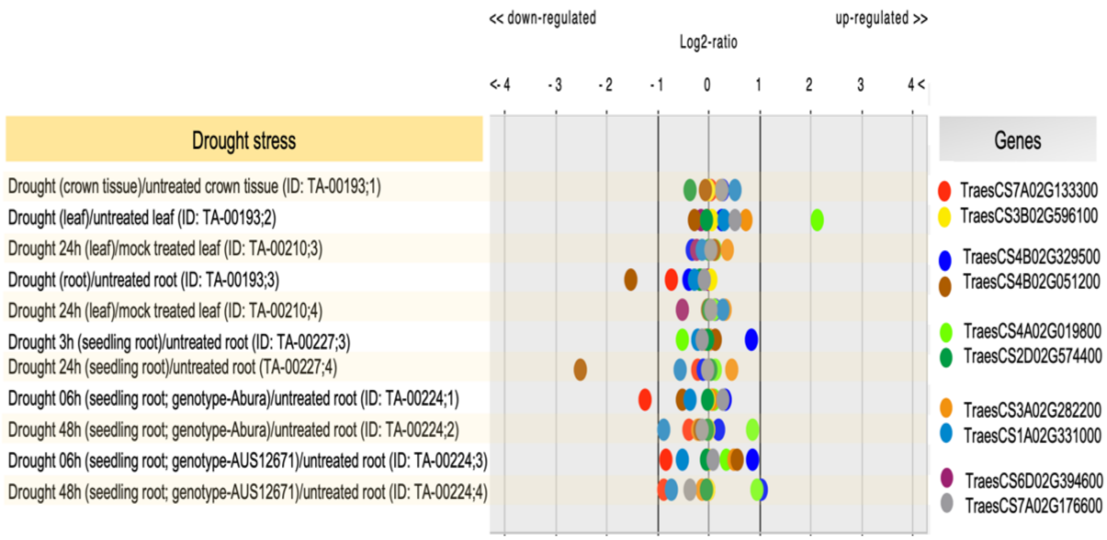

Supplementary Figure S6. Expression levels of the 10 candidate genes (CGs) after drought stress as shown by Genevestigator program.

Supplementary Tables

Supplementary Table S1. Details of the experimental sites, sowing dates, coordinates, mega-environment (ME), average maximum and average minimum (avg max/avg min) temperatures and total rainfall.

**Crop-season**	2011-12	2012-13
**Sites**	Meerut, India	Powarkheda, India
**Planting date**	Nov-16	Nov-21
**Coordinates**	28º.97'N 77º.74'E	22º.07'N 73º.98'E
**Altitude (m)**	218	299
**ME***	ME1	ME5
**Soil type****	Deep, clay soil	Deep, loamy soils
**Avg max/avg min temperature (°C)**	25.8/11.6	30.6/11.3
**Rainfall (mm)#**	73.7	63.8

*Source is CIMMYT wheat atlas, http://old.wheatatlas.org/; **NBSS & LUP, Nagpur, India; ME, mega-environments; Avg, Average; # Total rain-fall during the crop-season in mm

Supplementary Table S2. Descriptive statistics for four grain weight related traits in four environments. DTA, days to anthesis; DTM, days to maturity; GFD, grain filling duration; GNPE, grain number per ear; GWPE, grain weight per ear * E1, Meerut Irrigated; E2, Meerut rainfed; E3, Powarkheda irrigated; E4, Powarkheda rainfed.

Trait	Environment*	Range	Mean ± SE	Skewness	Kurtosis	CV (%)
DTA	E1	89.0 - 135.5	106.1 ± 0.7	0.8	-0.7	11.5
	E2	85.0 - 131.5	103.5 ± 0.6	0.7	-0.6	10.5
	E3	85.0 - 128	101.8 ± 0.5	0.9	-0.1	8.8
	E4	83.5 - 121	97.3 ± 0.5	0.8	-0.2	8.8
GFD	E1	24 - 37.5	32.7 ± 0.2	-0.7	-0.2	9.0
	E2	21.5 - 37	30.2 ± 0.2	-0.3	-0.1	9.2
	E3	23 - 39	32 ± 0.2	-0.7	0.5	8.9
	E4	15.5 - 37	28.9 ± 0.1	-0.5	2.2	8.9
GNPE	E1	18 - 66.7	43.7 ± 0.6	-0.1	-0.7	22.4
	E2	13.3 - 60.4	37 ± 0.5	0.0	-0.3	23.5
	E3	13.4 - 65.4	35 ± 0.6	0.1	-0.3	27.7
	E4	6.7 - 52.2	27.7 ± 0.4	0.3	0.3	26.4
GWPE	E1	0.4 - 3.2	1.6 ± 0	0.1	-0.5	34.0
	E2	0.3 - 2.5	1.2 ± 0	0.0	-0.7	37.2
	E3	0.2 - 2.7	1.5 ± 0	-0.1	-0.9	37.4
	E4	0.2 - 2.5	1.1 ± 0	0.5	0.9	34.7

Supplementary Table S3. List of significant SNP markers (qualified Bonferroni criteria) associated with seven different stress related indices (highlighted cells in orange colour) for each of the four traits at two locations (Meerut and Powarkheda). DI, drought resistance index; GMP, geometric mean productivity; MPI, Mean productivity index; RDI, relative drought index; STI, stress tolerance index; YI, yield index; YSI, yield stability index.

<table>
<tr><th rowspan="2">SNP/trait</th><th rowspan="2">Chr.;Pos</th><th colspan="7">Meerut</th><th colspan="7">Powarkheda</th></tr>
<tr><th>DI</th><th>GMP</th><th>MP</th><th>RDI</th><th>STI</th><th>YI</th><th>YSI</th><th>DI</th><th>GMP</th><th>MP</th><th>RDI</th><th>STI</th><th>YI</th><th>YSI</th></tr>
<tr><td colspan="16">Days to anthesis (DTA)</td></tr>
<tr><td>SNP_265</td><td>1A;167.84</td><td></td><td></td><td></td><td></td><td></td><td></td><td></td><td></td><td></td><td></td><td></td><td></td><td style="background:orange"></td><td></td></tr>
<tr><td>SNP_388</td><td>1A;229.05</td><td style="background:orange"></td><td></td><td></td><td></td><td></td><td></td><td></td><td></td><td></td><td></td><td></td><td></td><td></td><td></td></tr>
<tr><td>SNP_404</td><td>1A;247.88</td><td></td><td style="background:orange"></td><td style="background:orange"></td><td></td><td></td><td style="background:orange"></td><td></td><td></td><td></td><td></td><td></td><td></td><td style="background:orange"></td><td></td></tr>
<tr><td>SNP_647</td><td>1B;64.81</td><td></td><td style="background:orange"></td><td style="background:orange"></td><td></td><td style="background:orange"></td><td style="background:orange"></td><td></td><td></td><td></td><td></td><td></td><td></td><td></td><td></td></tr>
<tr><td>SNP_1116</td><td>1B;291.41</td><td></td><td></td><td></td><td style="background:orange"></td><td></td><td></td><td style="background:orange"></td><td></td><td></td><td></td><td></td><td></td><td></td><td></td></tr>
<tr><td>SNP_2283</td><td>2B;71.44</td><td></td><td></td><td></td><td></td><td></td><td></td><td></td><td></td><td></td><td></td><td></td><td></td><td style="background:orange"></td><td></td></tr>
<tr><td>SNP_2322</td><td>2B;95.62</td><td style="background:orange"></td><td></td><td></td><td></td><td></td><td></td><td></td><td></td><td></td><td></td><td></td><td></td><td></td><td></td></tr>
<tr><td>SNP_2800</td><td>2B;163.74</td><td></td><td></td><td></td><td></td><td></td><td></td><td></td><td style="background:orange"></td><td></td><td></td><td></td><td></td><td></td><td></td></tr>
<tr><td>SNP_2860</td><td>2B;179.51</td><td></td><td style="background:orange"></td><td style="background:orange"></td><td></td><td></td><td style="background:orange"></td><td></td><td></td><td></td><td></td><td></td><td></td><td></td><td></td></tr>
<tr><td>SNP_2981</td><td>2B;203.95</td><td style="background:orange"></td><td></td><td></td><td></td><td></td><td></td><td></td><td></td><td></td><td></td><td></td><td></td><td></td><td></td></tr>
<tr><td>SNP_3398</td><td>3A;75.97</td><td></td><td style="background:orange"></td><td style="background:orange"></td><td></td><td style="background:orange"></td><td style="background:orange"></td><td></td><td></td><td></td><td></td><td></td><td></td><td></td><td></td></tr>
<tr><td>SNP_4087</td><td>3B;103.66</td><td></td><td></td><td></td><td></td><td></td><td></td><td style="background:orange"></td><td></td><td></td><td></td><td></td><td></td><td></td><td></td></tr>
<tr><td>SNP_4482</td><td>3B;253.74</td><td></td><td></td><td></td><td></td><td></td><td></td><td></td><td></td><td></td><td></td><td></td><td></td><td style="background:orange"></td><td></td></tr>
<tr><td>SNP_5304</td><td>4B;60.12</td><td></td><td style="background:orange"></td><td style="background:orange"></td><td></td><td style="background:orange"></td><td></td><td></td><td></td><td></td><td></td><td></td><td></td><td></td><td></td></tr>
<tr><td>SNP_6054</td><td>5A;249.30</td><td></td><td></td><td></td><td></td><td></td><td></td><td></td><td></td><td style="background:orange"></td><td style="background:orange"></td><td></td><td style="background:orange"></td><td></td><td></td></tr>
<tr><td>SNP_7068</td><td>6A;88.94</td><td></td><td style="background:orange"></td><td style="background:orange"></td><td></td><td style="background:orange"></td><td style="background:orange"></td><td></td><td></td><td></td><td></td><td></td><td></td><td></td><td></td></tr>
<tr><td>SNP_7200</td><td>6A;161.18</td><td></td><td></td><td></td><td></td><td></td><td></td><td></td><td style="background:orange"></td><td></td><td></td><td></td><td></td><td></td><td></td></tr>
</table>

SNP_7816	6B;99.31															
SNP_7945	6D;25.79															
**Grain filling duration (GFD)**																
SNP_1444	1D;151.27															
SNP_1793	2A;135.42															
SNP_1804	2A;137.60															
SNP_2447	2B;134.76															
SNP_3008	2D;20.1051															
SNP_3105	2D;235.76															
SNP_3483	3A;89.84															
SNP_6185	5B;51.85															
SNP_6193	5B;52.21															
SNP_7541	6B;51.79															
SNP_8018	6D;186.19															
SNP_8312	7A;102.18															
SNP_8407	7A;145.43															
**Grain number per ear (GNPE)**																
SNP_388	1A;229.04															
SNP_659	1B;76.04															
SNP_1239	1B;428.18															
SNP_1410	1D;103.81															
SNP_1555	2A;34.05															
SNP_1962	2A;214.58															
SNP_2271	2B;64.70															
SNP_2359	2B;116.12															
SNP_2677	2B;152.09															
SNP_3074	2D;125.39															
SNP_3377	3A;60.57															
SNP_3768	3A;263.08															
SNP_3779	3A;268.05															
SNP_4116	3B;105.52															
SNP_4431	3B;199.80															
SNP_4743	3D;275.42															
SNP_4793	4A;7.22															
SNP_4925	4A;86.97															
SNP_5304	4B;60.12															
SNP_5704	5A;70.94															
SNP_5823	5A;96.99															
SNP_5883	5A;114.89															
SNP_6510	5B;153.64															
SNP_6940	6A;45.13															
SNP_7721	6B;65.33															
SNP_8047	6D;196.20															
SNP_8070	6D;210.93															
SNP_8220	7A;45.99															
SNP_8336	7A;114.95															
SNP_8603	7A;247.45															
SNP_9207	7B;184.42															
SNP_9322	7B;241.74															
**Grain weight per ear (GWPE)**																
SNP_73	1A;55.057															
SNP_404	1A;247.88															
SNP_436	1A;273.22															
SNP_829	1B;134.20															

SNP_1239	1B;428.18														
SNP_1829	2A;150.12														
SNP_2670	2B;151.35														
SNP_2841	2B;170.66														
SNP_3074	2D;125.39														
SNP_3377	3A;60.57														
SNP_3530	3A;96.63														
SNP_3831	3B;23.95														
SNP_4122	3B;107.36														
SNP_4179	3B;126.94														
SNP_4805	4A;23.05														
SNP_5003	4A;177.33														
SNP_5157	4A;227.26														
SNP_5215	4A;237.60														
SNP_6941	6A;45.13														
SNP_7068	6A;88.94														
SNP_7204	6A;161.18														
SNP_7732	6B;67.31														
SNP_7747	6B;69.052														
SNP_8047	6D;196.20														
SNP_8162	7A;28.43														
SNP_8239	7A;55.34														
SNP_8256	7A;77.39														
SNP_8418	7A;146.07														
